# Minocycline in chronic management of febrile infection-related epilepsy syndrome (FIRES): a case series and literature review of treatment strategies

**DOI:** 10.1186/s42494-025-00224-4

**Published:** 2025-06-06

**Authors:** Lanlan Feng, Hui Li, Lei Ma, Mengmeng Hu, Bo Hui, Zhongqing Sun, Xiaomu Wang, Yuanyuan Wang, Wen Jiang

**Affiliations:** Department of Neurology, Xijing Hospital, The Air Force Medical University, People’s Republic of China, No.127, Chang Le West Road, Xi’an, 710032 Shaanxi China

**Keywords:** FIRES, Minocycline, Drug-resistant epilepsy

## Abstract

The effectiveness of treatment for the chronic phase of febrile infection-related epilepsy syndrome (FIRES) remains uncertain. This study aimed to evaluate the therapeutic efficacy of minocycline in patients with chronic FIRES who had a poor response to conventional antiseizure medications. Three patients received 100 mg of minocycline (100 mg twice daily for 12 weeks), with effectiveness assessed based on seizure frequency, duration, type, and quality of life (using the quality of life in epilepsy-31, QOLIE-31), alongside adverse event monitoring. Results showed that one patient (Patient 3) exhibited a significant reduction in seizure duration and improved QOLIE-31 scores, with focal seizures being the only type observed after treatment. However, there was no statistically significant change in overall seizure frequency among the three patients. Additionally, a short literature review was conducted to explore various management strategies for chronic FIRES, including IL-1 receptor antagonist (anakinra) and IL-6 receptor antagonist (tocilizumab), centro-median thalamic nuclei deep brain stimulation, cannabidiol, responsive neurostimulation, intrathecal dexamethasone, ketogenic diet, and vagus nerve stimulation. In conclusion, considering the existing research on the etiological mechanisms of FIRES and based on our preliminary findings on the anti-inflammatory and antiepileptic properties of minocycline, early initiation of minocycline therapy in the chronic phase of FIRES should be explored further.

**Trial registration**

Clinicaltrials.gov (NCT05958069, retrospectively registered 22 July 2023).

## Background

Febrile infection-related epilepsy syndrome (FIRES) is characterized by the new-onset refractory status epilepticus (NORSE), which refers to the presence of a history of antecedent febrile infections within 1 to 14 days prior to the onset of refractory status epilepticus (RSE) in patients across the age range, regardless of fever presence during the seizure [1]. It has no recognisable acute or structural, toxic and metabolic causes [2, 3]. During the acute phase, there are no specific clinical manifestations and patients typically present with status epilepticus (SE) or frequent seizures accompanied by confusion. These conditions are often resistant to treatment with a combination of anti-seizure medications (ASMs) and may necessitate adjunctive interventions such as mechanical ventilation, high-dose intravenous phenobarbital, or midazolam [4]. Seizure activity may persist for up to 12 weeks during the acute phase, which is subsequently evolved into chronic phase [5]. Almost all patients during the chronic phase of FIRES developed drug-refractory epilepsy characterized by seizure forms similar to those observed in the acute phase, and this may be accompanied by cognitive decline, behavioral impairment, and psychiatric disorders [6, 7]. The quality of life in patients during the chronic phase is lower than that before the onset of the disease [8]. Therefore, the achievement of adjunctive therapeutic success is crucial in the chronic phase of the syndrome.

Although the etiology of FIRES remains entirely unclear, several studies have suggested that FIRES is caused by overactivation of the immune system after initial infection [1, 7, 9–11]. Following infection, pro-inflammatory cytokines are released activating innate immunity within the central nervous system. This process enhances the excitability of neurons, thereby increasing susceptibility to epilepsy. Meanwhile, status epilepticus can trigger pro-inflammatory processes and neuroinflammation, further exacerbating neuronal overexcitation and accelerating sustained epileptic seizures. Therefore, immunotherapy with corticosteroids and intravenous immunoglobulin (IVIg) is widely recommended in the acute phase, unfortunately, their response rates are often unsatisfactory [12]. The ketogenic diet emerges as a potentially treatment, but its effectiveness is not universally guaranteed [13]. Therefore, the chronic phase of FIRES presents significant therapeutic challenges. Although alternative treatments like cannabidiol, anakinra, rituximab, and epilepsy surgery have been considered in certain instances, few patients achieve seizure-free [14]. Nonetheless, few studies have assessed the impact of these interventions on patients with persistent drug resistant epilepsy (DRE) during the chronic phase of FIRES, leaving the efficacy of these approaches debated and uncertain [15, 16]. This persistent therapeutic gap underscores the urgent need for novel treatment modalities that directly target the underlying mechanisms of FIRES.

Minocycline is a stable and safe semi-synthetic tetracycline antibiotic with unique properties that may be allowed to cross the blood–brain barrier. It has also demonstrated anti-inflammatory, antioxidant, anti-apoptotic, and immunomodulatory effects [17, 18]. It has shown promising neuroprotective effects in various neurological studies such as traumatic brain injury, spinal cord injury, autoimmune encephalomyelitis, Alzheimer's disease, Parkinson's disease, focal and global ischemia, amyotrophic lateral sclerosis, and epilepsy [19]. In particular, it has emerged as potential treatment option for epilepsy due to its positive antiepileptic effects observed in in both animal experiments and clinical trials [20–22]. Patel et al. revealed that minocycline showed noticeable antiepileptic properties in a mouse model of seizures induced by the theiler murine encephalomyelitis virus (TMEV), which considerably reduced the frequency and severity of seizures [21]. Further studies have supported these findings, consistently reporting reductions in seizure severity, frequency, and duration in animal models of epilepsy related to TMEV infection [22–24]. Nowak et al. also demonstrated that minocycline could reduce seizure frequency and the incidence of DRE in patients with astrocytoma, a type of brain tumor [25]. However, there is a lack of data regarding its role during the chronic phase of FIRES. Here, we described the efficacy and safety of using minocycline in three patients with chronic phase of FIRES who failed conventional antiepileptic therapy. In addition, we conducted a literature review to explore the current treatment for the chronic phase of FIRES.

## Methods

### Participants

Three patients diagnosed with FIRES based on the 2018 expert consensus definition were included in this study [7]. These patients exhibited drug-resistant epilepsy during the chronic phase of FIRES [11]. The study was approved by the Clinical Research Ethics Committee of Xijing Hospital (XJLL-KY-20232108) and written informed consent was acquired. To explore a potential treatment regimen, minocycline was administered orally at 100 mg twice daily for 12 weeks, as per the recommendation of Food and Drug Administration [17]. During this period, patients continued with ASMs without any other treatment adjustments. The seizure type of patients, seizure frequency, seizure duration and quality of life via the quality of life in epilepsy-31 questionnaire (QOLIE-31) were monitored to assess the therapeutic efficacy. In addtion, the patients were asked to report any adverse events during the minocycline treatment.

### Data collection

Three patients were evaluated clinically neurological conditions using data collected from the Xijing Hospital Neurology Database prior to minocycline initiation. This study thoroughly documented the patients'profiles, including cerebrospinal fluid (CSF) assessments, electroencephalography (EEG), and magnetic resonance imaging (MRI) results. In addition, patients’ medical records were reviewed to extract acute-phase treatment protocols, seizure characteristics (frequency and type) and ASMs history. Patients were asked to record epilepsy diary and report adverse reactions as well as their QOLIE-31 scores both at baseline (4 weeks pre-treatment) and at 4-week intervals throughout the 12-week treatment period. The primary outcomes of the study was to determine whether there was a minimum 50% reduction in monthly seizure frequency after intervention. Secondary outcomes were defined as a reduction in seizure frequency and duration from baseline, as well as meaningful improvement in QOLIE-31 scores. Patients were considered treatment failures if they did not achieve the intended treatment goal or experienced a serious adverse event. The efficacy was described by comparing the outcomes with the baseline data.

### Literature review

**Fig. 1:**
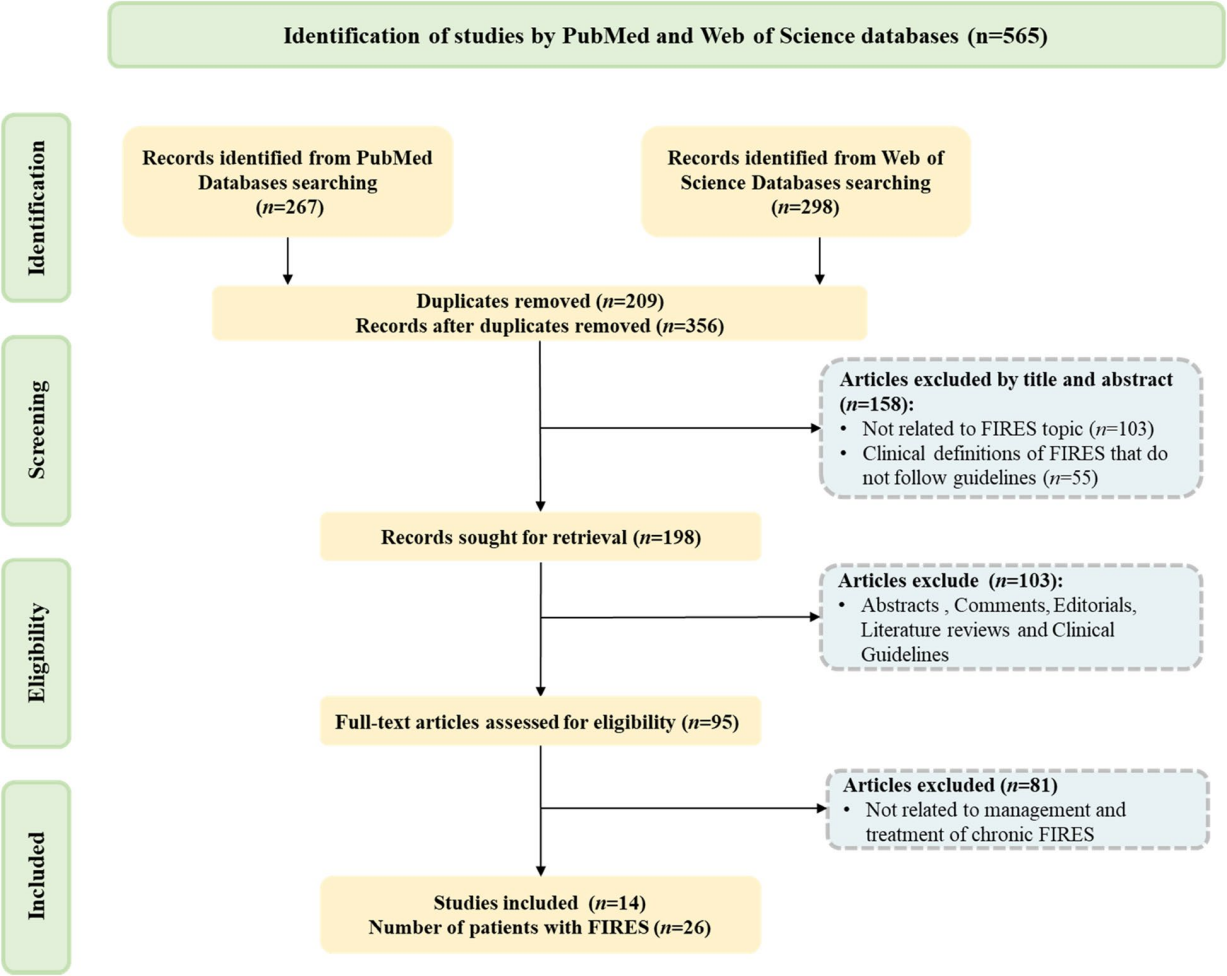
Flowchart of the literature on screening for the treatment of chronic FIRES in this study

We systematically searched the PubMed and Web of Science databases for articles published before September 2024, using the keywords"FIRES"or"febrile infection-related epilepsy syndrome". We removed duplicate articles to avoid repetition and filtered the results to retain only those directly relevant to our research topic. To further enriched our literature review, we also manually searched through the reference lists of selected articles, focusing on those that discussed treatment options for chronic FIRES. We limited our search to articles written in English and excluded studies that did not meet our specific criteria. Our exclusion criteria included the following: I) Articles not related to FIRES. II) FIRES diagnosis does not meet expert consensus [4]. III) Non-primary literature, including abstracts, comments, editorials, literature reviews, and clinical guidelines. IV) Studies not related to management and treatment of chronic FIRES. After a thorough evaluation of 565 articles, we identified and included 14 publications that met our stringent selection criteria (Fig. 1).

## Results

### Case description

#### Case 1

A 30-year-old female presented with a history of limb twitching and loss of consciousness following a brief fever, which had resolved with antipyretic medication. She had no prior history of epilepsy, relevant family history, or other neurological diseases. Upon admission, she underwent lumbar puncture for CSF metagenomic next-generation sequencing, autoimmune encephalitis-related antibody testing, and paraneoplastic antibodies screening. All results were negative except for a mild increase in CSF protein. Given her severe symptoms, including prolonged seizures (> 30 min) with loss of consciousness and hypoxia (PaO_2_), she was admitted to the intensive care unit (ICU). She received endotracheal intubation, ventilator support, along with antiviral, anti-infective, sedative, and antiseizure medications. From days 2–12, she was treated with first-line immunotherapy, including intravenous immunoglobulins and glucocorticoids, which led to an improvement in her symptoms. However, she faced challenges in weaning off the ventilator and continued experiencing frequent generalized convulsions. On days 16–17, her condition improved significantly after the adjustment of ASMs and discontinuation of propofol, enabling her successful transfer from the ICU. By day 45, she began to be gradually weaned from the ventilator, and her antiepileptic regimen was adjusted. By day 52, she was discharged with persistent generalized seizures (lasting 30–60 s, spontaneous frequency: 4–10 times/day). Despite continuous pharmacotherapy, the patient's seizures continued to recur during the 2-year follow-up period. Her chronic seizures primarily presented with generalized tonic–clonic seizures (GTCS) occurring every 1–2 days. Her EEG tests revealed multifocal seizure onset, and MRI showed bilateral hippocampal sclerosis with reduced hippocampal volume and increased signals in T2 WI FLAIR. In 2021 (two years after FIRES onset), she underwent transcranial magnetic stimulation (TMS), with no improvement in seizure control. In December 2022 (three years post-onset), she opted into this study and received minocycline as an adjunctive therapy.

#### Case 2

An 8-year-old boy was admitted to our hospital in June 2019 due to recurrent nocturnal focal epileptic seizures. On admission, his parents reported that he had a fever two days before admission, reaching a peak temperature of 38.4 °C. He demonstrated age-appropriate development with no history of neurological disorders. Initial treatment attempts with multiple antiseizure medications (sodium valproate, topiramate, oxcarbazepine, bilampanel, lacosamide, and clonazepam) failed to significantly reduce seizure frequency. Diagnostic investigations including physical examination, CSF analysis, and MRI revealed no significant abnormalities. On day 4 of his hospitalization, he received first-line immunotherapy (gamma globulin and high-dose glucocorticoid pulse therapy). This therapeutic intervention led to a noticeable improvement in his condition, and he was subsequently discharged on day 19. Nevertheless, during subsequent follow-up appointments, it was observed that while the frequency of his seizures had decreased, they still occurred 1 to 5 times per month. Additionally, he experienced speech loss and cognitive decline compared to peers. Over the subsequent two years of FIRES onset, despite trying an extensive array of ASMs (clonazepam, sodium valproate, levetiracetam, lamotrigine, topiramate, oxcarbazepine, bilampanel, lacosamide, and phenobarbital), the boy failed to achieve complete seizure control. His seizures were characterized as generalized tonic–clonic type with a increased frequency of approximately 15 episodes/month. His MRI scans revealed evidence of brain atrophy, and EEG recordings indicated that the origin of his epileptic discharges remained elusive in the chronic phase of his condition. He also attempted TMS treatment after two years of the FIRES onset with no improvement in seizure control. In July 2023, he opted to receive minocycline in an add-on treatment modality.

#### Case 3

A 17-year-old male patient was admitted to our hospital in January 2023 presenting fever-induced (at 39.5°C) recurrent focal seizures manifesting as twitching of the right mouth corner for 2–3 min, succeeded by eye deviation upwards, upper limb flexion, lower limb extension, urinary incontinence, and impaired consciousness. Each episode lasted for 40–60 s with interictal intervals of 30–40 s. Due to the persistence of status epilepticus (SE), he was transferred to the ICU, where he underwent intubation and was placed on mechanical ventilation. The patient received a comprehensive therapeutic regimen incorporating anti-infective, antiviral, sedative, and antiseizure medications. On admission (day 1), it showed no abnormalities in laboratory tests (CSF analysis and autoimmune antibody screening) and brain MRI, while EEG recordings revealed a predominant low-to-moderate amplitude alpha rhythm (7.5–8 Hz), with a propensity for occipital lobe activity, and diffuse low-amplitude beta activity across all leads. The patient responded positively to first-line immunotherapy with intravenous immunoglobulins and glucocorticoids from days 3 to 9, which led to a marked decrease in seizure frequency. By day 12, he was weaned off sedatives (midazolam and propofol) and successfully extubated. On day 15, he was transitioned to the general ward for ongoing management with a tailored ASMs regimen comprising lacosamide, levetiracetam, and clonazepam. On day 22, the patient was discharged, albeit with persistent seizures characterized by eye rolling, limb twitching, right-sided mouth twitching, and brief loss of consciousness, each episode lasting approximately 2 min. The seizures persisted at a frequency of 5–8 episodes per day, being precipitated by visual auras, such as photopsia with star-like patterns, and sometimes preceded by brief involuntary movements of the upper limbs for 10–40 s. At the 60-day follow-up, there was no further improvement, and neuroimaging remained stable. In April 2023 (day 100 post-admission), the patient consented to participate in our study and was administered minocycline as an adjunctive therapy.

Table 1 summarized the clinical profiles of all three enrolled.

**Table 1:** Clinical characteristics of participants in the acute and chronic phases

Patient	Age at FIRES	Sex	Acute phase CSF	Acute phase therapy	Frequency of episodes in the chronic phase	Forms of seizures	EEG in the chronic phase	NMRI	ASMs	Other treatments in the chronic phase
1	30y	F	Elevated protein	GC IVIg Propofol Midazolam	5.25 times/w	GTCS	Multifocal onset	LHS	LEV, LTG, TPM, PB, VPA	TMS
2	8y	M	N	GC IVIg	3.75 times/w	GTCS	Unidentified origin	Brain atrophy	LEV, LTG, PB, VPA	TMS
3	18y	M	N	GC IVIg Midazolam	10.25 times/w	GTCS, FMS	Multifocal onset	N	LEV, LCM, PHT, Clonazepam	-

*y* Year, *F* Female, *M* Male, *CSF* Cerebrospinal fluid, *N* Normal, *GC* Glucocorticoids, *IVIg* Intravenous immunoglobulins, *times/w* times/week, *GTCS* Generalized tonic clonic seizure, *FMS* Focal motor seizures, *EEG* Electroencephalography, *NMRI* Nuclear magnetic resonance imaging, *LHS* Lateral hippocampal sclerosis, *ASMs* anti-seizure medication, *LEV* Levetiracetam, *LTG* Lamotrigine, *TPM* Topiramate, *PB* Phenobarbital, *VPA* Sodium valproate, *LCM* Lacosamide, *PHT* Phenytoin, *TMS* Transcranial magnetic stimulation

### Outcomes

The median interval from the acute phase of FIRES to the minocycline intervention was 12 months (range:3–36 months). All patients received a 12-week course of adjunctive minocycline while maintaining their previous ASMs with no new anticonvulsant therapies introduced in the preceding 3 months. Despite completing the full 12-week course, none of the three patients experienced a significant reduction in seizure frequency. Nevertheless, Patient 3 demonstrated notable improvements, including a mean reduction of 76% in mean seizure duration, and seizure types from two subtypes to focal motor seizures at the study endpoint. QOLIE-31 scores increasing from 31 at baseline to 49 after 12 weeks of treatment, particularly in cognitive and psychosocial domains.

Regarding treatment safety, Patient 1 briefly experienced side effects with anorexia and nausea after 55 days of minocycline therapy, both of which resolved spontaneously within 24 h. The other two patients tolerated the medication well with no reported adverse effects.

**Fig. 2:**
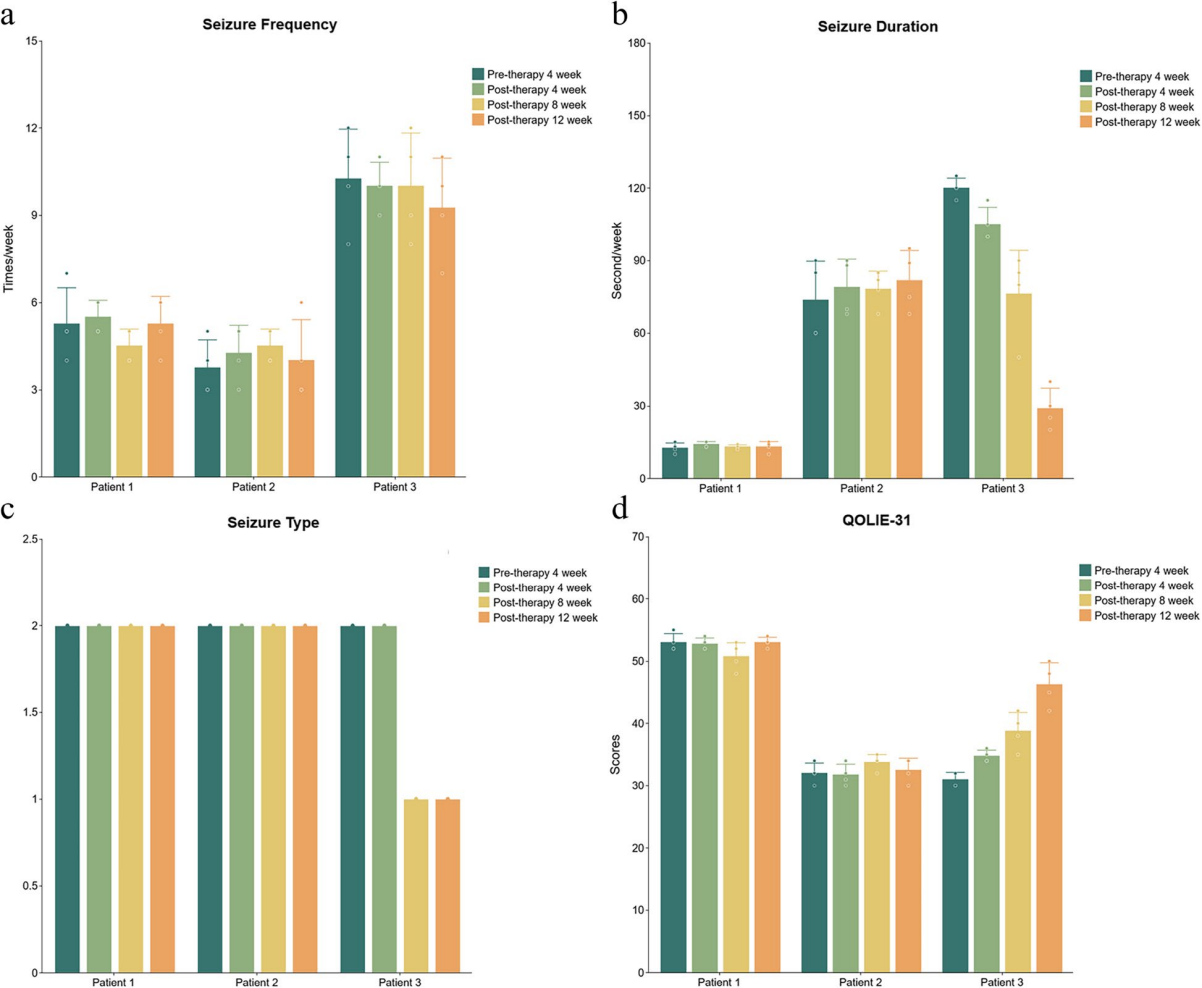
The outcomes of seizure frequency, seizure duration, seizure type, and QOLIE-31 scores at weeks 4, 8, and 12 during minocycline treatment with respect to their baseline conditions are presented for the three patients with chronic FIRES. **a** Seizure frequency; **b** Seizure duration; **c** Seizure type; **d** QOLIE-31 scores. Bars represent the average value, and error bars indicate standard error. Dots indicate weekly measurements for each individual

All patients underwent systematic monitoring: baseline assessments were recorded 4 weeks before treatment initiation, followed by repeated evaluations every 4 weeks during the 12-week intervention. Detailed data on the follow-up of three patients receiving 12-week minocycline treatment was presented in Fig. 2.

### Literature review

This review ultimately included 14 articles on various treatment options for the chronic phase of FIRES [15, 16, 26–37]. There are five studies on IL-1 receptor antagonists (anakinra) and one case involving tocilizumab [15, 16, 26, 28, 29], one article on intrathecal dexamethasone (IT-DEX) [30], two studies on cannabidiol (CBD) [31, 32], three studies on ketogenic diet (KD) [33–35], one article on deep brain stimulation of the centromedian thalamic nuclei (CMN-DBS) combined with anakinra [27], one study on responsive neurostimulation (RNS) [36], and one article on vagus nerve stimulation combined with cannabidiol (VNS/CBD) [37].

Anakinra is commonly used in the treatment of autoinflammatory diseases, with several studies corroborating its effectiveness in the chronic phase of FIRES. Kenney-Jung et al. first administered anakinra in pediatric patients across the acute to chronic phase of FIRES. During the chronic phase, anakinra demonstrated robust tolerability and exhibited significant efficacy in mitigating seizures associated with FIRES [26]. Furthermore, three independent case reports demonstrated that anakinra exhibited significant reductions in seizure frequency and severity in FIRES patients who were unresponsive to conventional antiseizure medications and immunomodulatory therapy [15, 28, 29]. Fortunately, one case achieved complete seizure remission, and treatment also appeared to enhance cognitive and behavioral outcomes [28, 29]. However, the case series study conducted by Aledo-Serrano et al. demonstrated that the therapeutic effect of anakinra is not universally effective [16]. The researchers evaluated seizure outcomes, non-seizure complications, and adverse events among the five patients, revealing that 3 of 5 patients experienced a moderate reduction (20–50%) in seizure frequency after six months of anakinra treatment. For patient in the cohort who was insensitive to anakinra and subsequently switched to tolizumab, a comparable 30% reduction in seizure frequency was observed at the end of treatment [16]. The use of anakinra/tolizumab suggested that an anti-inflammatory approach may be beneficial in the management of FIRES. By targeting the interleukin-1 pathway, anakinra may modulate the neuroinflammatory processes that contribute to the persistence of epilepsy in FIRES [26]. This is supported by evidence of normalization of pro-inflammatory cytokine levels in the cerebrospinal fluid of treated patients. Anti-inflammatory therapy may disrupt the cycle of seizure-induced neuroinflammation, thereby preventing long-term neurological sequelae [26]. Future studies should focus on determining the optimal timing and duration of anakinra therapy in FIRES, identifying the subgroup of patients who would benefit most from anakinra treatment. The efficacy of tocilizumab in FIRES underscores the importance of anti-inflammatory therapy, particularly in regions where anakinra and tocilizumab lack approval for neurological indications [30]. In such settings, Horino et al. reported successful treatment with IT-DEX in the chronic phase of FIRES. Thiopental sodium was successfully discontinued, on day 3 after the IT-DEX treatment initiation, with no further clinical seizures observed over the 17-month follow-up, but the patient's EEG examination showed persistent epileptic discharges in the temporal and frontal lobes at the last follow-up [30]. This study also suggested significantly elevated CSF levels of pro-inflammatory cytokines (e.g. CXCL10, CXCL9, IFN-γ, IL-1β, IL-6, and IL-8) in the CSF of the patients compared to the controls (patients with chronic epilepsy) prior to IT-DEX treatment. Notably, CXCL10 and neopterin levels decreased significantly after treatment but remained elevated compared to controls, suggesting that inflammation was suppressed but not fully resolved by IT-DEX [30]. In the chronic phase of FIRES, CBD was also used as an add-on treatment in an open-label case series. Gofshteyn et al. observed significant reductions in seizure frequency and duration in the five patients [31]: seizure frequency reduced by an average of 90.9% at 4 weeks and 65.3% at 48 weeks after starting CBD. However, some patients experienced side effects such as dizziness, loss of appetite, weight loss, and nausea/vomiting during the treatment, though these might not have been directly attributable to CBD. Vicino et al. conducted a retrospective observational study to assess the efficacy and safety of CBD as an add-on treatment for drug-resistant epilepsy. It included a patient with FIRES who had previously failed to respond to multiple ASMs. Following CBD initiation, the patient experienced a reduction in seizure frequency [32]. Due to several confounding factors in these studies, including the lack of standardised controls for duration, dose and cycle of cannabidiol treatment, and the limited follow-up periods, the efficacy of CBD as an add-on therapy in the chronic phase of FIRES needs to be further validated and explored. Three children received KD in the chronic phase of FIRES, with two-thirds experiencing positive outcomes [33]. Similarly, in another study, two children with chronic FIRES achieved seizure freedom after KD treatment during follow-up periods of 18 and 20 months [34]. In addition, adult male patient with a 27-month history of FIRES exhibited a 75% reduction in seizure frequency and mild cognitive improvement after KD initiation, although he experienced concomitant weight loss [35]. However, the complexity of KD may affect long-term patient compliance.

Finally, recent research has illuminated the clinical significance of neuromodulatory treatment modalities in FIRES. These modalities included CMN-DBS combined with anakinra, RNS, and VNS paired with CBD, all of which have exhibited promising antiepileptic efficacy. Sa et al. revealed that two FIRES patients who were resistant to multiple ASMs received CMN-DBS treatment achieved complete cessation of generalized seizures. After the administration of anakinra, one patient experienced a transient seizure remission lasting three weeks [27]. These findings further support the hypothesis that neuroinflammation may be a contributing factor in FIRES. Theroux et al. described a case involving a child who developed refractory multifocal epilepsy subsequent to FIRES and was treated with RNS. The RNS system successfully detected and terminated the seizures, reducing their seizure frequency from 1–4 times/week to 0–4 times/month over an eight-month treatment period. Additionally, cognitive improvements were also observed in the patient [36]. Bonardiet al. reported on a FIRES patient treated with VNS in conjunction with CBD and multiple ASMs. The treatment led to rapid ceasation of status epilepticus and maintained seizure-freedom during the chronic phase of FIRES [37]. However, it is important to note that the concurrent administration of other treatments (such as anakinra and CBD) in the aforementioned studies necessitates caution in isolating the impact of each individual treatment. While generally well-tolerated, neuromodulation therapies remain risks of complications, including infection and equipment failure. Therefore, ongoing monitoring and careful consideration of potential risks are essential in the application of these treatments. Detailed information about the treatment courses and outcomes of the above studies were presented in Table 2.

**Table 2:** Details and results of other treatment-related studies in the chronic phase of FIRES

References	Population (Adult/Child)	Number of patients, Sex (F/M)	Number of anti-seizure medications in chronic phase (pre)	Other treatment of chronic phase	Treatment began close to the onset of FIRES	Dose/Duration	Adverse events	Number of anti-seizure medications in chronic phase (post)	Follow-up outcome	Seizure free
Kenny-Jung et al. (2016) [26]	Child	1	NA	Anakinra	6 days	200 mg/day on hospital day 6–23, 54–190 and day 191-end	No	NA	Frequent clinical seizures on week seven of the wean anakinra (receiving once-daily dosing) Full dose anakinra was reinitiated and patients remained without clinical seizures for the next six weeks	No
Westbrook et.al (2019) [28]	Adult	1	5	Anakinra	31 days	100 mg/3 times/day on hospital day 32; 100 mg/2 times/day on hospital day 48 100 mg/day on hospital day 64	No	5	Seizures ceased within 24 h No seizures and regaining baseline physical and cognitive abilities at 6-month outpatient follow-up	Yes
Dilena et al. (2019) [15]	Child	1 (NA)	6	Anakinra	18 months	100 mg/day; 7 months	No	3	Seizure reduction (21 (mean) to 1 (mean) times) Seizures and EEG epileptiform abnormalities increased after anakinra with drawal	No
Cupane et al. (2023) [29]	Child	1 (1/0)	4	Anakinra	36 months	50 mg/day; 1 year	No	4	Seizure reduction (21 to 4 times/day) Cognitive and motor dysfunction improvement	No
Aledo-Serrano et al. (2022) [16]	Child	5 (3/2)	8.3	Anakinra/Tocilizumab	7 years (2,11) (median)	Anakinra: 100–200 mg/day; 9 months (median) Tocilizumab (8 mg/kg every 4 weeks); 9 months (median)	No	3.8	The 3/5 patients reported reduced seizure frequency (20–50%), seizure intensity, rescue medication needed, and better behavior/communication	No
Horino et al. (2021) [30]	Child	1 (0/5)	11	IT-DEX	59 days	0.15–0.25 mg/kg/day, 4cyclical	NA	4	Outcome at discharge from hospital: Seizure frequency-annually Outcome at the last follow-up (17 months): seizure free	Yes
Gofshteyn et al. (2017) [31]	Child	5 (NA)	7 (5,9) (median)	Cannabidiol	3 months (3,81) (median)	25 mg/kg/day	Dizziness appetite and weight decreased Nausea/vomiting	3 (1.5,3.25) (median)	The average seizure counts decreased by 90.94% after 4 weeks post-cannabidiol The average seizure counts decreased by 71.8% after 48 weeks post-cannabidiol	1/5
Walter et al. (2023) [38]	-	1 (NA)	NA	Cannabidiol	NA	20 mg/kg/day Underwent VNS implantation in the same period	NA	NA	The seizures frequency was temporarily reduced	NA
Caraballo et al. (2013) [33]	Child	3 (NA)	NA	KD	NA	NA	NA	NA	2/3patients had sporadic seizures (brief seizures every 2 and every 4 months) after 2 and 10 years of follow-up	NO
Singh et al. (2014) [34]	Child	2 (1/1)	3	KD	13/3 days	Patient 1: KD with a 4:1 fat-to-carbohydrate and protein ratio, initiated at 50% of daily caloric needs and titrated over 5 days to reach 100%. 3 weeks later, KD with 3.25:1; over 20 months Patient 2: KD with 6:1 fat-to-carbohydrate and protein ratio; over 4 months	NA	3/2	Seizure free	Yes
Obara et al. (2022) [35]	Adult	1 (0/1)	6	KD	27 months	The patient with a 2:1 (fat: carbohydrate and protein) ratio KD of 1,350 kcal The patient was then administered a 1.5:1 ratio KD of 1,800 kcal due to a weight decreased by 10 kg since KD initiation at 36 months after the onset	Weight decreased	4	The seizures had reduced by > 75%, the behavioral problems gradually subsided, and the attention and durability during rehabilitation improved at three-month follow-up after the KD initiation	No
Sa et al. (2019) [27]	Child	2 (0/2)	NA	CMN-DBS/Anakinra	27/37 days	Patient-1: CMN-DBS was implanted and stimulation commenced at 4 mA, frequency 130 Hz, and pulse with 90 mcs at 27 days. Anakinra: 5 mg/kg/day over 14 days Patient-2: Anakinra was started on 22 days and titrated up to 10 mg/kg/day CMN-DBS was implanted and bilateral neuromodulation commenced on day 37 at 2 mA/130 Hz/90 mcs at 27 days	No	NA	Patient-1: CMN-DBS was switched off 8 months after implantation and this was not followed by clinical deterioration or increase in seizure frequency The patients receiving Anakinra after 15 months had short focal seizures with an average of 2–5 seizures per month Patient-2: No improvement in consciousness and seizure activity	No
Theroux et al. (2020) [36]	Child	1 (0/1)	6	RNS	28 days	The RNS neurostimulator was placed in the left front oparietal region and connected to one left orbitofrontal depth lead and one left superior temporal cortical strip lead	NA	6	Over 8 months, clinical seizures decreased in frequency, from 1–4 seizures perweek preimplantation to 0–4 seizures per month, and in severity, with less frequent generalized convulsions	NA
Bonardi et al. (2023) [37]	Child	1	6	VNS/CBD	43 days	The VNS was implanted and CBD was administered, 43 days and 70 days, respectively	No	6	The patient was 8-months seizure free on multiple ASMs, talked properly and fluently, and resumed home schooling at twelve-month follow-up	No

*FIRES* Febrile infection-related epilepsy syndrome, *EEG* Electroencephalography, *CMN-DBS* Centro median thalamic nuclei deep brain stimulation, *CBD* Cannabidiol, *RNS* Responsive neurostimulation, *IT-DEX* Intrathecal injection of dexamethasone, *KD* ketogenic diet, *ASMs* Antiseizure medications, *VNS* Vagas nerve stimulation

Currently, standardized treatment protocols for the chronic phase of FIRES remain significantly lacking. This gap stems from multiple factors, including substantial variations in medication dosage, treatment duration, and overall therapeutic course, coupled with discrepancies in the criteria for evaluating therapeutic outcomes, thereby leading to complexity and inconsistency in assessing therapeutic efficacy. Furthermore, even when employing the same treatment modality, substantial variations in efficacy and considerable inter-individual variability are observed, which further amplifies the complexity and uncertainty surrounding treatment. Current understanding of chronic-phase FIRES treatment remains constrained by a paucity of high-quality evidence, with available data predominantly limited to case reports and small case series. There is a lack of large-scale, multi-centre, randomized controlled trials to substantiate the effectiveness of various treatments. Given the long-term and ongoing nature of chronic phase treatment, due consideration of treatment-related side effects and safety concerns is imperative. Specifically, the delicate balance between efficacy and safety must be meticulously evaluated when selecting treatment options. Notably, neuroinflammation occupies a pivotal role in the pathogenesis of FIRES. Consequently, future research endeavors should be focused on the development of innovative and anti-inflammatory agents with enhanced efficacy and improved safety profiles. These therapeutic agents would ideally modulate specific inflammatory pathways or cytokines implicated in FIRES, ultimately achieving better seizure control through precise immunomodulation.

## Discussion

While recent studies on FIRES have focused on acute-phase management, data on chronic-phase treatment remain limited. In this study, we evaluated adjunctive minocycline therapy in three patients with chronic FIRES. During the 12-week follow-up, Patient 3 demonstrated a significant improvement in seizure duration (now limited to focal seizures), along with enhanced quality of life (QOLIE-31 score increase). However, minocycline did not significantly reduce the seizure frequency in any of the patients.

This result may be attributed to the earlier initiation of minocycline treatment in Patient 3 (administered closer to the acute phase) compared to Patient 1 and Patient 2. Research suggests that early intervention with minocycline may be more effective in alleviating inflammation [27], potentially elucidating the observed clinical improvement and further supporting the notion that minocycline's anticonvulsant effects may mitigate the inflammatory storm. Therefore, the anti-inflammatory effects of minocycline are likely to be more pronounced in the early chronic phase, aiding in the reduction of intracranial inflammation and the improvement of clinical symptoms. Imaging reports of Patient 1 revealed hippocampal sclerosis, while Patient 2 exhibited cerebral atrophy. Minocycline appears to provide no benefit for these two patients with structural imaging changes. This may be because early administration of minocycline is more effective in preventing subsequent damage when imaging shows no significant permanent brain damage or structural alterations. However, for patients already demonstrating hippocampal sclerosis and cerebral atrophy, the efficacy of minocycline may be limited, as these irreversible structural changes are likely unresponsive to treatment. This perspective suggests that irreversible structural brain damage may be a negative predictor in the effectiveness of minocycline in FIRES patients. Therefore, the decision to incorporate minocycline into treatment should account for both imaging characteristics and clinical presentation [39].

In this study, we also documented the safety of minocycline in the treatment of FIRES. Patient 1 experienced mild gastrointestinal symptoms, including nausea and anorexia, on day 55 of the minocycline administration. These adverse effects were transient, resolving within 1–2 days, and did not recur during subsequent clinical evaluations. Although minocycline is sometimes associated with gastrointestinal upset, such reactions are more commonly observed in early phase of treatment [40]. Additionally, it is pertinent to note that Patient 1 was taking concomitant ASMs, which themselves may contribute to gastrointestinal disturbances [41]. The remaining two patients in this series report no adverse effects during the treatment phase. Overall, the tolerability profile of minocycline in this context appears to be favorable. Thereafter, we will continue to closely monitoring of these three patients and strive to obtain the necessary EEG and MRI to fully evaluate the efficacy of minocycline in the chronic phase of FIRES. It is important to note that the clinical application of minocycline in FIRES and other neurological disorders requires further validation through rigorous clinical trials involving a large number patients.

The therapeutic mechanism of minocycline in FIRES needs to be fully explored. The antiepileptic effects of minocycline are primarily attributed to its anti-inflammatory and immunomodulatory effects. Specifically, minocycline inhibits the activity of matrix metalloproteinases (MMPs), which play a role in immune responses. Minocycline exerts its anti-inflammatory effects by inhibiting various pathways, including those involving nuclear factor kappa-light-chain-enhancer of activated B cells (NF-κB), lipopolysaccharide-induced TNF-α factor (LITAF), nuclear hormone receptor (Nur77), lipopolysaccharide (LPS)-induced lectin-like oxidized low-density lipoprotein receptor-1 (LOX-1), phosphoinositide 3-kinase/Akt (PI3 K/Akt) pathway, and p38 mitogen-activated protein kinase (MAPK) activation. Minocycline attenuated microglia-T cell interactions via selective NFAT inhibition, implicating NFAT-mediated signaling as a therapeutic target in neuroinflammatory pathways and down-regulating CD40 ligands in T cells to minimize CD4 (+) T cell activation [17, 19, 42], potentially reducing inflammation. In addition to its effects on immune cells, minocycline has been found to alleviate depressive behavior in the rat model of chronic temporal lobe epilepsy by decreasing the levels of pro-inflammatory cytokines (IL-6 and IL-1 β) in the hippocampus [43]. In the analysis of CSF cytokine profiles, patients diagnosed with FIRES exhibited a pronounced upregulation of specific cytokines and chemokines. This inflammatory signature is notably distinct from that observed in patients with chronic epilepsy or non-inflammatory neurological disorders who present with frequent seizures [10] suggesting a FIRES-specific pathophysiological mechanism. Although the role of minocycline in treating DRE is relatively well-characterized from clinical and preclinical studies, there is a significant gap in research directly exploring the effects of minocycline in animal models of FIRES. The lack of such investigations hinders our capacity to establish a clear correlation between minocycline treatment and clinical outcomes in FIRES, highlighting the need for further targeted research in this area.

The potential etiology of FIRES involves multifactorial triggers, including non-specific systemic infections and/or genetic susceptibilities that lead to aberrant innate immune activation, ultimately driving neuroinflammation and epileptogenesis [1]. Cytokine profiling monitoring suggests that pro-inflammatory cytokines/chemokines, such as IL-6, TNF-α, CXCL8/IL-8, CXCL9, CXCL10,CXCL11,CCL2, CCL19,MIP-1α, and IL-12p70 are abnormally elevated in both serum and CSF of FIRES patients. These elevated cytokine/chemokine patterns correlate with disease pathogenesis, suggesting their direct involvement in sustaining neuroinflammatory processes and promoting seizure generation [10, 44]. Lin et al. proposed a hypothesis that excessive activation of the microglial NLRP3 inflammasome/IL-1 axis is a key initiatory event in FIRES based on a comprehensive literature analysis. This hyperactivation is thought to establish a neuroinflammatory environment that promotes seizure activity. The reciprocal relationship between inflammation and seizures is proposed to form a vicious cycle, thereby perpetuating the disease state. Additionally, the distinctive properties of microglia are hypothesized to enhance unopposed IL-1 signaling, resulting in chronic sterile neuroinflammation [45]. In a mouse model of transient middle cerebral artery occlusion (tMCAO) ischemic stroke, minocycline has been demonstrated to exert neuroprotective effects. Investigators discovered that minocycline administration significantly diminished levels of interleukin-1 beta (IL-1β) and interleukin-18 (IL-18) by suppressing the activation of the NLRP3 inflammasome within microglial cells. These cytokines, IL-1β and IL-18, are typically upregulated in response to ischemic injury and act as pivotal mediators of the NLRP3 inflammasome [46]. Consequently, the modulation of NLRP3 in FIRES could be a promising therapeutic target for minocycline intervention.

Currently, there are no specific treatments for chronic-phase of FIRES. Most patients required long-term management with ASMs and/or a combination of other therapies, including cannabidiol, VNS, and even epilepsy surgery. However, these treatments have shown limited efficacy in managing the chronic phase of FIRES. Our study aimed to contribute to the understanding of the chronic phase of FIRES by providing insights to guide future studies. Given the role of immune abnormalities and neuroinflammation in FIRES, treatments targeting these underlying mechanisms may represent a promising therapeutic avenue. There is increasing focus on immunomodulatory approaches, including steroids, IVIg, plasma exchange, and immunosuppressive agents. Moreover, the comprehensive management of the chronic phase of FIRES goes beyond just controlling seizures. It also involves proactive rehabilitation efforts aimed at improving the overall quality of life for patients.

## Conclusions

In summary, the chronic phase of FIRES remains a significant challenge, requiring both innovative therapeutic strategies and comprehensive care. This small case series highlights observations of improved outcomes associated with minocycline treatment, suggesting potential benefits in mitigating disease progression. While these preliminary findings underscore the promise of timely anti-inflammatory interventions like minocycline, further research and clinical validation are essential to confirm their efficacy and optimize therapeutic protocols for FIRES management.

## Data Availability

The datasets of the current study are available from the corresponding author on reasonable request.

## Abbreviations

ASMs: Anti-seizure medications
CBD: Cannabidiol
CMN-DBS: Deep brain stimulation
CSF: Cerebrospinal fluid
EEG: Electroencephalography
FIRES: Febrile infection-related epilepsy syndrome
GTCS: Generalized tonic-clonic seizures
ICU: Intensive care unit
IT-DEX: Intrathecal dexamethasone
IVIg: Intravenous immunoglobulin
KD: Ketogenic diet
MRI: Magnetic resonance imaging
NORSE: New-onset refractory status epilepticus
QOLIE-31: Quality of life in epilepsy-31
RNS: Responsive neurostimulation
RSE: Refractory status epilepticus
SE: Status epilepticus
TMEV: Theiler murine encephalomyelitis virus
TMS: Transcranial magnetic stimulation
VNS: Vagus nerve stimulation
